# CT-based body composition and its change through time in relation to outcomes in participants screened for lung cancer

**DOI:** 10.1016/j.ebiom.2026.106276

**Published:** 2026-04-30

**Authors:** Stijn Bunk, Edwin Bennink, Grigory Sidorenkov, Marjolein A. Heuvelmans, Harry J.M. Groen, Hester A. Gietema, Mathias Prokop, Joachim G. Aerts, Colin Jacobs, Geertruida H. de Bock, Pim A. de Jong, Rozemarijn Vliegenthart, Firdaus Mohamed Hoesein, NELSON-POP consortium, Joachim G. Aerts, Robin Cornelissen, Ralph Stadhouders, Jeroen G.J. van Rooij, Lianne Trap, Mathias Prokop, Cornelia Schaefer-Prokop, Colin Jacobs, Geertruida H. de Bock, Marjolein A. Heuvelmans, Grigory Sidorenkov, Danrong Zhong, Harry J.M. Groen, Rozemarijn Vliegenthart, Pim A. de Jong, Firdaus A.A. Mohamed Hoesein, Stijn Bunk, George S. Downward

**Affiliations:** a University Medical Center Utrecht, Utrecht University, Utrecht, the Netherlands; b University Medical Center Groningen, University of Groningen, Groningen, the Netherlands; c Maastricht University Medical Center, Maastricht University, Maastricht, the Netherlands; d Radboud University Medical Center, Nijmegen, the Netherlands; e University Medical Center Rotterdam, Erasmus University Rotterdam, Rotterdam, the Netherlands

**Keywords:** Radiology, Thorax, Tomography, Computed, Body composition, Mortality, Lung cancer

## Abstract

**Background:**

Computed Tomography (CT) scans allow opportunistic evaluation of body composition. We investigated whether body composition and change through time are associated with lung cancer incidence and all-cause/lung cancer-specific mortality in a lung cancer screening cohort.

**Methods:**

A machine learning segmentation method was used in this retrospective cohort study to measure skeletal muscle area and density, and subcutaneous adipose tissue area (SAT) on repeated chest CTs from the Dutch-Belgian lung cancer screening trial. Hazard ratios by sex adjusted for age, smoking status, and smoking pack-years (aHR) were calculated for each outcome.

**Findings:**

During median follow-up of 12.2 (interquartile range, 1.2) years, 4.1% of 6187 subjects (85.5% male, mean age ± SD, 58.6 ± 5.5 years, smoking pack-years 41.2 ± 18.3) developed lung cancer, 12.2% died, and 2.1% died due to lung cancer. For males, SAT loss was associated with lung cancer incidence (aHR 1.19, 95% CI 1.02–1.39) and lung cancer-specific mortality (aHR 1.26, 95% CI 1.03–1.55), and less baseline muscle and muscle loss with all-cause mortality (aHR 1.20, 95% CI 1.10–1.31 and 1.17, 1.07–1.27). For females, less baseline SAT and SAT loss was associated with all-cause mortality (aHR 1.44, 95% CI 1.06–1.97 and 1.48, 1.13–1.94) and lung cancer-specific mortality (aHR 2.85, 95% CI 1.50–5.39 and aHR 1.96, 1.11–3.44). Models improved by including body composition trends for all-cause mortality (males: p < 0.001; females: p = 0.012) and for lung cancer-specific mortality (males: p = 0.102; females: p = 0.005).

**Interpretation:**

Body composition trends based on automated analysis of chest CT are associated with worse outcomes in participants screened for lung cancer.

**Funding:**

Dutch Cancer Society, Health Holland, Siemens Healthineers.

**Research in context**

**Evidence before this study**

Previous studies have shown that body composition is related to outcomes in persons who smoke(d) and in persons with cancer. This relationship is most often investigated based on a single time-point body composition measurement. Lower amounts of muscle and fat and lower muscle radiodensity (indicating lower muscle quality) were found to be related to increased all-cause mortality and lung cancer-specific mortality. However, little information exists on how longitudinal trends in body composition are related to outcomes.

**Added value of this study**

In the NELSON lung cancer screening trial, most subjects underwent low-dose chest CT at least 4 times over on average five years. We used a machine learning segmentation method to measure the body composition of NELSON subjects on these CT scans at multiple time points. Using these multiple measurements, we were able to calculate the longitudinal trends (change in measurement per year) in body composition. Low baseline measurements of muscle area, muscle radiodensity, and fat area as well as loss of muscle and loss of fat over time were related to increased mortality and lung cancer incidence. These relationships differed between men and women.

**Implications of all the available evidence**

This study revealed previously unknown relationships between longitudinal trends in body composition and outcomes in lung cancer screening subjects. Knowledge of the relative risk of lung cancer and mortality could allow for personalisation of lung cancer screening intervals. Future studies are needed to evaluate possible effects of personalised screening intervals.

## Introduction

Prevention of adverse outcomes in people who smoke(d) is a major challenge. To date, lung cancer screening has primarily focussed on pulmonary nodules and their associated risk of malignancy and mortality.^1^, ^2^, ^3^ However, smoking, the main risk factor for lung cancer development, has systemic effects on other organs and body composition as well.^4^, ^5^, ^6^ Body composition is a potentially modifiable factor in addition to smoking cessation. Changing body composition by lifestyle modifications may have a beneficial effect on outcomes.^7^

There is a wide variety of mechanisms by which body composition can affect outcomes in persons who smoke(d) and in those with cancer.^8^ Numerous studies have evaluated the relation of single time-point body composition measurements with outcomes. Firstly, in patients with lung cancer, body composition measurements based on chest computed tomography (CT) were related to lung cancer-specific mortality.^9^^,^^10^ Secondly, both the cross-sectional area (amount) and radiodensity (a proxy for muscle quality, where lower density indicates myosteatosis) of skeletal muscle and area (amount) of subcutaneous fat (SAT) were found to be associated with increased mortality among individuals with non-small cell lung cancer.^11^ Thirdly, cachexia was associated with increased mortality in small-cell lung cancer.^12^ Fourthly, the quantity and radiodensity of skeletal muscle could predict the incidence and progression of lung cancer, and all-cause mortality.^13^^,^^14^ Finally, detailed measurements of body composition were found to be more strongly associated with outcomes than the body mass index (BMI).^15^^,^^16^ In the lung cancer screening setting specifically, two studies investigated the relationship of body composition with lung cancer incidence based on baseline low-dose chest CT scans.^10^^,^^14^ One of those studies found an association between body composition, specifically low pectoralis muscle area, and increased lung cancer incidence risk.^14^

Currently, most studies relating body composition to mortality are based on a single baseline measurement, but longitudinal evaluation of trends in body composition could provide additional information on the risk of adverse outcomes. So far, research that has investigated the value of repeated evaluation of body composition focused on patients who already received a cancer diagnosis. For example, in colorectal cancer, a reduction in skeletal muscle over time was linked to disease progression.^17^ A setting in which repeated CT scanning is performed, allowing for determining the trend in body composition, is lung cancer screening. In the National Lung Screening Trial (NLST), a high-risk population was screened for lung cancer by low-dose chest CT. In that population, two-year trends in epicardial adipose tissue volume and radiodensity were found to be associated with all-cause mortality, whilst an increase in epicardial adipose tissue radiodensity was associated with increased lung cancer-specific mortality.^18^ To our knowledge, no study has investigated the value of repeated body composition evaluation in a lung cancer screening setting over a period longer than two years.

Therefore, in a lung cancer screening cohort screened over a period of on average five years, we determined baseline values and trends in body composition using Artificial Intelligence (AI) based automated body composition assessment on repeated low-dose chest CT scans. We established the association of these body composition parameters with the risk of three outcomes; all-cause mortality, lung cancer incidence, and lung cancer-specific mortality. We hypothesised that not only lower baseline body composition values for skeletal muscle and subcutaneous fat but also a decrease in the amount of muscle and subcutaneous fat and in muscle radiodensity are associated with worse outcomes.

## Methods

### Study population

The current retrospective cohort study is part of the NELSON-POP project, which aims to optimise participant selection and nodule management in lung cancer screening using a multimodal data approach.^19^ The data used in this retrospective study was obtained from the prospective NELSON trial. The NELSON trial included individuals aged 50–75 years, at the University Medical Centre Groningen, the University Medical Centre Utrecht, the Kennemer Gasthuis Haarlem, all in the Netherlands, and the University Hospitals Leuven, Belgium. Included participants were current or former smokers with a smoking history of >15 cigarettes/day during >25 years or >10 cigarettes/day during >30 years. Detailed in- and exclusion criteria of the NELSON trial have previously been described.^20^, ^21^, ^22^ Participants of the NELSON trial were randomised into two groups: a control group in which no lung cancer screening was performed and a screening group that underwent multiple low-dose chest CT scans in up to four screening rounds.^23^ The NELSON trial screening rounds were conducted from April 2004 through March 2012.^23^^,^^24^ Follow-up was conducted up to 2016. The current study comprised Dutch participants from the screening group only. Additional inclusion criteria for this study were a minimum of two CT-scans per subject with a minimum of 800 days between the first scan and last scan (without interval diagnosis of lung cancer) to measure the trends in body composition.

### Ethics statement

The NELSON trial complied with the regulations set out in the Wet bevolkingsonderzoek (WBO). On December 23, 2003, the Minister of Health of the Netherlands approved randomisation of persons to the NELSON trial. On March 22, 2006, the trial was retrospectively registered in Het Overzicht van Medisch-wetenschappelijk Onderzoek in Nederland (OMON) (NL-OMON22971). Written informed consent was obtained from all participants.

### Outcome determination and clinical factors

Subjects with positive screening tests were referred to a chest physician for workup and diagnosis.^21^ If lung cancer was diagnosed, the participant was treated for the disease and left the screening trial.^24^ No further CT scans were included for these participants. Data on work-up, cancer diagnosis and stage, treatment, vital status, and cause of death were obtained through linkages with the Dutch Centre for Genealogic and Heraldic Studies, Statistics Netherlands, and the Dutch Cancer Registry. For participants suspected to have died of lung cancer, cause of death was determined by an independent committee.^25^ Time to death and time to lung cancer diagnosis were defined as the time between the date of randomisation and the date of event. The date of randomisation was defined for each subject separately based on when the subject was randomised into the screening group of the NELSON trial.

Age, smoking status, and pack-years were all defined at date of randomisation on the basis of a questionnaire. Age and pack-years were continuous variables, whilst smoking status was split into two categories, currently smoking and not currently smoking.

### Image acquisition

Non-contrast low-dose chest CT scans were obtained in inspiration with 16-slice multidetector CT systems with collimation of 16 × 0.75 mm (Sensation-16, Siemens Medical Solutions, Forchheim, Germany; M × 8000 IDT, and Brilliance 16P, Philips Medical Systems, Cleveland, OH, USA). The settings used were fixed 30 mAs with 120 kVp for participants weighing 80 kg or less and 140 kVp for those weighing more than 80 kg. Medium-soft reconstruction kernels were used to reduce noise. Data were reconstructed as axial slices of 1 mm at 0.7 mm increment.^21^^,^^26^^,^^27^

### Body composition measurement

Obtaining body composition measurements from the chest CT scans required careful selection of a processing method. Due to the significant time required to manually segment a dataset of this scale, an automated deep-learning based segmentation approach was chosen. The segmentation method selection process was described previously.^28^ In short, CT scans, collected in a lung cancer screening context, have inconsistent fields-of-view (FOV) across the various screening rounds. This inconsistency had to be compensated for, as a reduction of or positional shift in FOV can lead to truncation of the body, which would subsequently yield longitudinal inconsistencies in measured body composition. Thus, we selected an open-source body composition calculation method called S-EFOV (Semantically Extended Field Of View), which generates plausible image data outside the originally acquired FOV.^29^^,^^30^ This method measures the area and density of skeletal muscle and area of subcutaneous fat at the vertebral levels T5, T8, and T10. This method achieved a mean ± Standard Deviation (SD) Dice Similarity Coefficient of 0.97 ± 0.03 on 2657 scans from the National Lung Screening Trial and Vanderbilt Lung Screening Program. In short, the segmentation process identifies the T5, T8 and T10 slices, imputes missing parts of the body outside the field-of-view, segments the skeletal muscle and subcutaneous fat, and calculates area and radiodensity.^29^^,^^30^ Measurements of area and density of skeletal muscle and area of subcutaneous fat were obtained for all CT scans of all subjects.

**Fig. 1:**
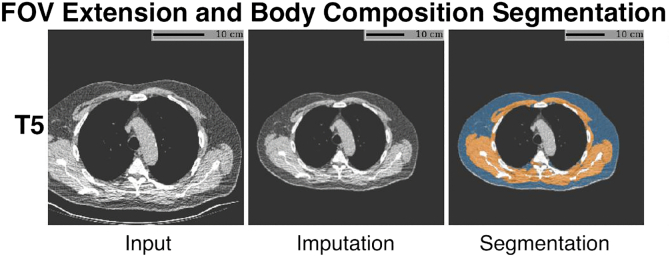
Segmentation process example. The input image shows the initial CT image. In this image part of the subcutaneous fat and part of the muscle is missing on the right side of the body. The imputation image shows the newly generated ‘complete’ image data. The segmentation image shows the segmentation generated based on the Imputation image. Orange = Skeletal muscle. Blue = Subcutaneous adipose tissue. For the skeletal muscle both area and density is computed.

In existing literature, the level just above the aortic arch is a common measuring point in thoracic body composition.^31^, ^32^, ^33^, ^34^ Therefore, we analysed the T5 level. An example of the segmentation process can be seen in Fig. 1.

**Fig. 2:**
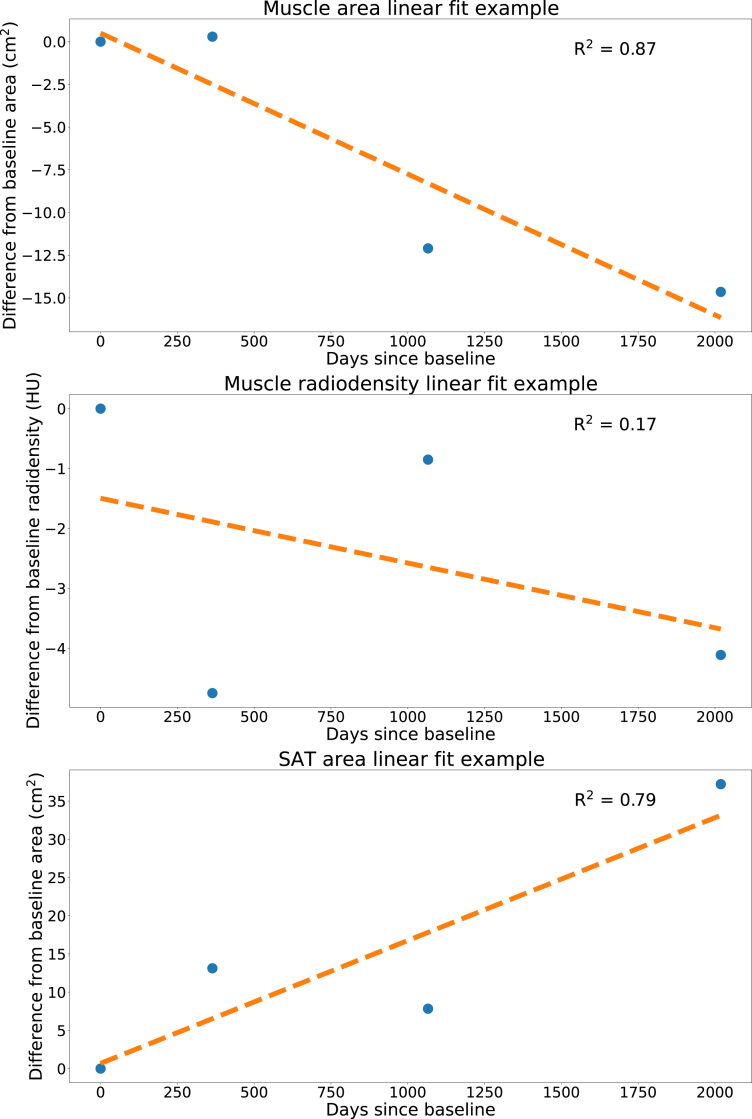
Examples of three linear fits for an individual screening participant. The top plot shows a decreasing muscle area trend with a low spread of the measurement points. The middle plot shows a decreasing muscle radiodensity trend with a large spread of the measurement points. The bottom plot shows an increasing subcutaneous fat area trend with a low spread of the measurement points.

To obtain interpretable per subject metrics for longitudinal rate of change in body composition we used ordinary least squares linear regression models. We defined the first scan as zero days since baseline and then for each subsequent scan determined the number of days between the date of the first scan and the date of the specific subsequent scan, yielding for each subject a list of body composition values at specific days since baseline. The trend (rate of change in Δ/365 days) was calculated by applying an ordinary least squares linear regression fit to this list of body composition values and days since baseline for each participant. Based on visual inspections of the fitted linear regression models and evaluation of the R^2^ statistics least squares linear regression models proved to be an appropriate representation of the data. Some example fits are shown in Fig. 2.

### Statistical analysis

Normally distributed continuous variables are reported as means ± SDs, non-normally distributed as median (interquartile range, IQR). Categorical variables are reported as numbers of participants and their percentage of the total. Separate analyses were performed for males and females. For the analysis, we used the baseline body composition values alongside the body composition trend as variables in a Cox regression model, as well as three standard factors, age, smoking status, and pack-years. We analysed three different outcomes, namely all-cause mortality risk, lung cancer incidence risk, and lung cancer-specific mortality risk. No variables violated the proportional hazard assumption. We obtained adjusted hazard ratios (aHRs) for all-cause mortality, lung cancer incidence, and lung cancer-specific mortality for baseline values of skeletal muscle area and its trend, skeletal muscle density and its trend, and subcutaneous fat area and its trend. The statistical analysis was performed using R 4.4.1 with the package *survival*.^35^, ^36^, ^37^ We used p < 0.05 to indicate statistical significance. False discovery rate adjustment was performed with the Benjamini-Hochberg procedure. We grouped our statistical tests by input variable, yielding m = 6. The aHRs are reported with their 95% confidence interval in parentheses. For the body composition variables, aHRs are reported per standard deviation decrease of each variable. To investigate the added value of body composition measurements for Cox regression modelling, we used the likelihood ratio test (LRT) to compare nested models including additional variables. The first model included only the standard factors, the second model included the standard factors and the baseline body composition, and the third model included the standard factors, the baseline body composition, and the body composition trends. We performed the LRT between the first and second model, and the second and third model.

### Role of funders

The funders had no role in study design, data collection, data analyses, interpretation, or writing of report.

## Results

### Population characteristics

**Fig. 3:**
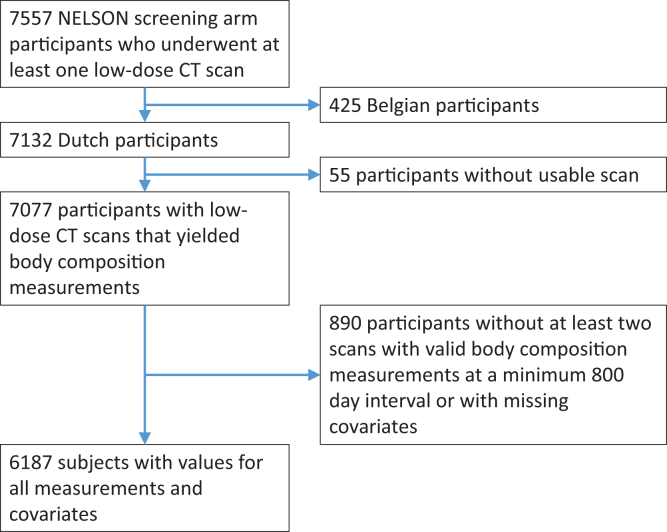
Subject selection flowchart. Only analyses based on the 6187 subjects are presented in this paper.

A total of 6187 individuals were included in the analysis (age 58.6 ± 5.5 years) of which 5228 (85.5%) were male (Fig. 3). Most people (4,560, 73.5%) had four or more CT-scans obtained over a period of 5.0 ± 1.1 years. Population and body composition characteristics are summarised in Table 1. As of the last follow-up date in 2016, with median 12.2 (IQR 1.2) years of follow-up since inclusion, 757 subjects died (12.2%), 256 subjects were diagnosed with lung cancer (4.1%), and 128 died of lung cancer (2.1%). Median follow-up was shorter for subjects who died versus those who did not, 8.9 (IQR 3.8) years versus 12.2 (IQR 0.7) years for males and 9.3 (IQR 3.2) years versus 11.4 (IQR 0.2) years for females. On average, across the population, there was a decreasing trend for muscle area and density and increasing trend for SAT area over time. The muscle area linear regression fits had a median R^2^ of 0.41 (IQR 0.64), muscle density a median R^2^ of 0.60 (IQR 0.63), and SAT area a median R^2^ of 0.55 (IQR 0.65). An evaluation of potential non-linear hazards is reported in Appendix A. Details on the number of CT-scans per subject are summarised in Appendix B. An evaluation of segmentation error rates is reported in Appendix C. Population characteristics of the included and excluded participants are reported in Appendix D.

**Table 1:** Population characteristics, overall and by sex.

Characteristic	Overall (n = 6187)	Male (n = 5228)	Female (n = 959)
Age (year)	58.6 ± 5.5	58.8 ± 5.4	57.8 ± 5.5
Currently smoking, n (%)	3408 (55.1%)	2864 (54.8%)	544 (56.7%)
Pack-years	41.2 ± 18.3	41.3 ± 18.6	40.6 ± 16.4
Follow-up years (IQR)	12.2 (1.2)	12.2 (1.0)	11.4 (0.2)
Deaths, n (%)	757 (12.2%)	685 (13.1%)	72 (7.5%)
Lung cancer cases, n (%)	256 (4.1%)	219 (4.2%)	37 (3.9%)
Lung cancer deaths, n (%)	128 (2.1%)	109 (2.1%)	19 (2.0%)
Baseline to final scan time (years)	5.0 ± 1.1	5.0 ± 1.1	4.9 ± 1.1
Baseline muscle area (cm^2^)	217.2 ± 38.0	228.2 ± 29.2	157.1 ± 20.2
Trend of muscle area (cm^2^/year)	−1.7 ± 3.9	−1.8 ± 4.1	−0.9 ± 3.0
Baseline muscle density (HU)	28.4 ± 5.6	29.0 ± 5.5	25.7 ± 5.4
Trend of muscle density (HU/year)	−0.5 ± 0.8	−0.5 ± 0.8	−0.4 ± 0.7
Baseline subcutaneous fat area (cm^2^)	162.7 ± 73.7	152.3 ± 64.1	219.5 ± 93.8
Trend of subcutaneous fat area (cm^2^/year)	2.4 ± 7.8	2.3 ± 7.1	3.1 ± 10.8

Data given are mean ± standard deviation of number and percentage. HU = Hounsfield Unit.

### Adjusted hazard ratios of outcomes

Details on the adjusted hazard ratios are reported in Table 2. We found significant associations of body composition and body composition trends with all-cause mortality, lung cancer incidence, and lung cancer-specific mortality. For males, age, smoking status, and pack-years were highly significant independent risk factors for all outcomes (p < 0.002). However, for females, age was not a significant risk factor for lung cancer incidence and lung cancer-specific mortality (p = 0.49 and 0.97, respectively), smoking status was not significantly associated with lung cancer incidence (p = 0.21), and pack-years not with lung cancer incidence (p = 0.066). Given that risks differed between males and females, stratified analyses are presented. All adjusted hazard ratios are presented per standard deviation difference in the variable.

**Table 2:** Adjusted hazard ratios for the three proportional hazard models.

Variables	aHR per SD/Cat	All-cause mortality		Lung cancer incidence		Lung cancer-specific mortality	
		aHR	p-value	aHR	p-value	aHR	p-value
Age							
M	5.4 years	1.50 (1.39, 1.62)	<0.001^a^	1.28 (1.12, 1.47)	<0.001^a^	1.35 (1.11, 1.64)	0.002^a^
F	5.5 years	1.34 (1.09, 1.67)	0.006^a^	1.11 (0.82, 1.52)	0.49	0.99 (0.64, 1.53)	0.97
Current smoking							
M	smoking	1.55 (1.32, 1.82)	<0.001^a^	1.70 (1.27, 2.28)	<0.001^a^	2.16 (1.41, 3.30)	<0.001^a^
F	smoking	2.27 (1.31, 3.92)	0.003^a^	1.61 (0.76, 3.38)	0.21	6.85 (1.41, 33.2)	0.017^a^
Smoking history							
M	18.6 pack-years	1.15 (1.08, 1.23)	<0.001^a^	1.24 (1.11, 1.38)	<0.001^a^	1.31 (1.13, 1.52)	<0.001^a^
F	16.5 pack-years	1.24 (1.01, 1.53)	0.037^a^	1.28 (0.98, 1.68)	0.066	1.50 (1.07, 2.12)	0.020^a^
Subcutaneous fat, baseline							
M	−64.1 cm^2^	1.03 (0.93, 1.14)	0.59	1.37 (1.14, 1.65)	<0.001^a^	1.27 (0.99, 1.63)	0.06
F	−93.8 cm^2^	1.44 (1.06, 1.97)	0.020^a^	2.12 (1.36, 3.32)	<0.001^a^	2.85 (1.50, 5.39)	0.001^a^
Subcutaneous fat, loss/year							
M	−7.1 cm^2^/year	1.06 (0.97, 1.15)	0.21	1.19 (1.02, 1.39)	0.028^a^	1.26 (1.03, 1.55)	0.028^a^
F	−10.8 cm^2^/year	1.48 (1.13, 1.94)	0.005^a^	1.29 (0.88, 1.90)	0.2	1.96 (1.11, 3.44)	0.019^a^
Muscle, baseline							
M	−29.2 cm^2^	1.20 (1.10, 1.31)	<0.001^a^	1.01 (0.87, 1.18)	0.85	0.88 (0.71, 1.09)	0.24
F	−25.7 cm^2^	0.96 (0.73, 1.25)	0.74	0.74 (0.51, 1.07)	0.11	0.54 (0.32, 0.92)	0.023
Muscle, loss/year							
M	−4.1 cm^2^/year	1.17 (1.07, 1.27)	<0.001^a^	0.94 (0.81, 1.11)	0.49	1.03 (0.83, 1.28)	0.8
F	−3.0 cm^2^/year	1.13 (0.90, 1.44)	0.3	1.32 (0.95, 1.82)	0.1	1.64 (1.07, 2.52)	0.024
Muscle density, baseline							
M	−5.5 HU	1.27 (1.15, 1.40)	<0.001^a^	1.38 (1.15, 1.64)	<0.001^a^	1.53 (1.19, 1.96)	<0.001^a^
F	−5.4 HU	1.60 (1.18, 2.18)	0.003^a^	1.84 (1.19, 2.84)	0.006^a^	2.26 (1.18, 4.33)	0.014^a^
Muscle density, decrease/year							
M	−0.8 HU/year	1.11 (1.01, 1.22)	0.031	1.16 (0.98, 1.38)	0.076	1.13 (0.90, 1.43)	0.29
F	−0.7 HU/year	1.19 (0.91, 1.57)	0.2	1.06 (0.73, 1.53)	0.78	0.88 (0.54, 1.46)	0.63

All models include all the variables listed in the variables column, and therefore all hazard ratios are adjusted for all other variables. One model relates the variables to all-cause mortality, one relates them to lung cancer incidence, and one relates them to lung cancer-specific mortality. The adjusted hazard ratios are reported per step or category in the column labelled aHR per SD/Cat. Smoking status is categorical, all other variables are continuous. For smoking status the adjusted hazard ratio is reporting relative to no smoking. For the age and pack-years the adjusted hazard ratios are reported per one standard deviation increase. For the body composition values the adjusted hazard ratios are reported per one standard deviation decrease. The adjusted hazard ratios for smoking status indicate the hazard of smoking versus no smoking and for the continuous variables indicate how much the hazard changes if the measurement changes by the value in aHR per SD/Cat column. The standard deviations are the ones reported in Male and Female columns of Table 1. Values between parentheses are the 95% CI hazard ratios. aHR = Adjusted Hazard Ratio. SD = Standard Deviation. Cat = Category. HU = Hounsfield Unit.

a p < 0.05 after adjustment for multiple comparisons with the use of the Benjamini-Hochberg procedure (m = 6).

### All-cause mortality

For males, low baseline muscle area (aHR 1.20 per SD, 95% CI 1.10–1.31, p < 0.001) and low baseline muscle density (aHR 1.27, 1.15–1.40, p < 0.001) were associated with increased risk of all-cause mortality. Independent of the baseline body composition, loss of muscle area (aHR 1.17, 1.07–1.27, p < 0.001) and decreasing muscle density (aHR 1.11, 1.01–1.22, p = 0.031) were associated with increased risk of all-cause mortality, although for decreasing muscle density this relation was not significant after Benjamini-Hochberg correction. Baseline subcutaneous fat area and loss of subcutaneous fat area were not associated with all-cause mortality.

For females, low baseline subcutaneous fat area (aHR 1.44, 1.06–1.97, p = 0.020) and low baseline muscle density (aHR 1.60, 1.18–2.18, p = 0.003) were associated with increased risk of all-cause mortality. Independent of the baseline body composition, loss of subcutaneous fat area (aHR 1.48, 1.13–1.94, p = 0.005) was associated with increased risk of all-cause mortality. Baseline muscle area, loss of muscle area, and decreasing muscle density were not associated with all-cause mortality.

### Lung cancer incidence

For males, low baseline subcutaneous fat area (aHR 1.37, 1.14–1.65, p < 0.001) and low baseline muscle density (aHR 1.38, 1.15–1.64, p < 0.001) were associated with increased risk of lung cancer incidence. Independent of the baseline body composition, loss of subcutaneous fat (aHR 1.19, 1.02–1.39, p = 0.028) was associated with increased risk of lung cancer incidence. Baseline muscle area, loss of muscle area, and decreasing muscle density were not associated with lung cancer incidence.

For females, low baseline subcutaneous fat area (aHR 2.12, 1.36–3.32, p < 0.001) and low baseline muscle density (aHR 1.84, 1.19–2.84, p = 0.006) were associated with increased risk of lung cancer incidence. Loss of subcutaneous fat area, baseline muscle area, loss of muscle area, and decreasing muscle density were not associated with lung cancer incidence.

### Lung cancer-specific mortality

For males, low baseline muscle density (aHR 1.53, 1.19–1.96, p < 0.001) was associated with increased risk of lung cancer-specific mortality. Independent of the baseline body composition, loss of subcutaneous fat area (aHR 1.26, 1.03–1.55, p = 0.028) was associated with increased risk of lung cancer-specific mortality. Baseline subcutaneous fat area, baseline muscle area, loss of muscle area, and decreasing muscle density were not associated with lung cancer-specific mortality.

For females, low baseline subcutaneous fat area (aHR 2.85, 1.50–5.39, p = 0.001) and low baseline muscle density (aHR 2.26, 1.18–4.33, p = 0.014) were associated with increased risk of lung cancer-specific mortality. Low baseline muscle area (aHR 0.54, 0.32–0.92, p = 0.023) was associated with reduced risk of lung cancer-specific mortality, though this relation was not significant after Benjamini-Hochberg correction. Independent of the baseline body composition, loss of subcutaneous fat area (aHR 1.96, 1.11–3.44, p = 0.019) and loss of muscle area (aHR 1.64, 1.07–2.52, p = 0.024) were associated with increased risk of lung cancer-specific mortality, though for loss of muscle area this relation was not significant after Benjamini-Hochberg correction. Decreasing muscle density was not associated with lung cancer-specific mortality.

### Added value of body composition and body composition trends

Compared to basic Cox regression models that only included age, smoking status, and pack years, models including those variable alongside metrics for baseline body composition showed improved goodness of fit based on the LRT for all-cause mortality for males (χ^2^= 37.23, p < 0.001) but not for females (χ^2^= 6.92, p = 0.075), lung cancer incidence for males (χ^2^= 14.00 p = 0.003) and females (χ^2^= 12.34, p = 0.006), and lung cancer-specific mortality for males (χ^2^= 10.96, p = 0.012) and females (χ^2^= 11.79, p = 0.008).

Including all previous variables as well as body composition change through time in the models improved the goodness of fit based on the LRT for all-cause mortality for males (χ^2^= 26.10, p < 0.001) and females (χ^2^= 11.01, p = 0.012), and lung cancer-specific mortality for females (χ^2^= 11.90, p = 0.008) but not for males (χ^2^= 6.20, p = 0.102). Additionally, for lung cancer incidence the goodness of fit was not improved for males (χ^2^= 5.66, p = 0.129) or females (χ^2^= 5.15, p = 0.161). Details can be found in Table 3.

**Table 3:** Added value of body composition to the model for risk determination of all-cause mortality, lung cancer incidence, and lung cancer-specific mortality.

	All-cause mortality	Lung cancer incidence	Lung cancer-specific mortality
LRT between covariates only and with addition of baseline body composition			
M			
χ^2^	37.23	14.00	10.96
p-value	<0.001	0.003	0.012
F			
χ^2^	6.92	12.34	11.79
p-value	0.075	0.006	0.008
LRT between covariates with baseline body composition and with addition of body composition trends			
M			
χ^2^	26.10	5.66	6.20
p-value	<0.001	0.129	0.102
F			
χ^2^	11.01	5.15	12.86
p-value	0.012	0.161	0.005

Likelihood Ratio Tests (LRT) were performed using one-way analysis of variance. All models include the covariates age, smoking status, and pack-years.

## Discussion

In a large prospective cohort of males and females with a substantial smoking history who participated in lung cancer screening, we investigated CT-based body composition parameters and especially the body composition trends over time and their relation to all-cause mortality, lung cancer incidence, and lung cancer-specific mortality. According to our hypothesis we observed that not only low baseline measurements but also downward trends in body composition biomarkers were related to higher risk of all-cause mortality, lung cancer incidence, and lung cancer-specific mortality. We also found differences in the risk relationships between males and females. A key sex-specific difference was that lower baseline muscle area was significantly associated with increased all-cause mortality in males, whereas lower subcutaneous fat area was significantly associated with all-cause mortality in females.

Most previous research assessing body composition via automated machine learning methods used CT measurements taken at a single time point. Our hazard ratios for baseline measurements align with results from previous studies. Specifically, a prior study using the same FOV-extending segmentation method in 20,768 participants from the National Lung Screening Trial demonstrated that cross-sectional muscle and fat measurements provided independent predictive value for all-cause mortality and lung cancer death.^10^ This matches our findings, although we also found that low baseline fat area was associated with an increased risk of lung cancer incidence, which the prior study did not demonstrate. In another study it was reported that low muscle area and quality, and low subcutaneous fat area were associated with increased risk of all-cause mortality in people with non-small cell lung cancer, which is consistent with our findings.^11^ Additionally, it was reported in a study conducted in a large lung cancer screening cohort of 1696 participants that an association exists between low pectoral muscle area and increased lung cancer incidence.^14^ This finding contrasts with our results, as we did not observe a relationship between skeletal muscle area and lung cancer incidence. Potential explanations for these discrepancies may include differences in study population, differences body composition measurement techniques, particularly the use of automated machine learning body composition measurement algorithms, and differences in statistical methodologies.

Previous research on repeated analysis of body composition, on subsequent CT scans through time, is limited and has primarily focused on epicardial adipose tissue, different from subcutaneous fat as in our research. A significant relationship between decreased epicardial adipose tissue volume and increased risk of all-cause mortality and lung cancer-specific mortality was reported.^18^ These findings are consistent with our observed associations between loss of subcutaneous fat and increased risk of all-cause and lung cancer-specific mortality. Although subcutaneous fat, unlike visceral fat, is sometimes not considered a major risk factor, our results show that subcutaneous fat loss is associated with adverse outcomes. We do not expect our inclusion criteria to have reduced the generalisability of our findings on repeated analysis of body composition to similar lung cancer screening cohorts, provided those other cohorts followed a similar screening procedure, and the measurements are performed in a similar manner.

The median R^2^ values of around 0.5 mean there is variance in the longitudinal body composition data that an ordinary least squares linear model does not capture. Our goal was to obtain interpretable subject-specific rate of change measurements for use in hazard models to determine if changes in body composition have an effect on outcomes, for which the remaining unexplained variance was not a problem. However, the high unexplained variance means that application of the hazard models on individuals would introduce uncertainty in the risk estimations. Therefore, in the future, other models of body composition change may be required to more accurately model individual risk.

Sex-specific differences in risk associations of interest as well. For all-cause mortality, muscle quality was a relevant factor in both males and females. However, baseline muscle area was specifically associated with risk in males, while subcutaneous fat area was relevant in females. The underlying causes of these sex-specific discrepancies remain unclear and warrant further investigation. We also found that a greater number of body composition metrics were associated with lung cancer incidence in males, whereas more metrics were associated with lung cancer-specific mortality in females. Notably, among females, lower baseline muscle area was associated with a decreased risk of lung cancer-specific mortality, an unexpected finding. However, this association did not remain significant after controlling for false-positives with the Benjamini-Hochberg procedure, suggesting it was likely a false-positive result.

To assess the added value of body composition measures, likelihood ratio testing was performed. The inclusion of baseline muscle area, muscle density, and subcutaneous fat area significantly improved the fit of Cox regression models beyond models including only age, smoking status, and pack-years, except in the case of all-cause mortality among women, where no significant improvement was observed. This exception may reflect weaker risk associations for baseline measures in women compared with men. The most important observation was that the inclusion of longitudinal body composition trends significantly improved model fit for all-cause mortality and lung cancer-specific mortality in women, and for all-cause mortality in men. However, no improvement in model fit was observed for lung cancer incidence in either sex. Nevertheless, in men, loss of subcutaneous fat area was significantly associated with an increased risk of lung cancer incidence. The lack of improvement in model fit for lung cancer incidence is likely attributable to the fact that loss of subcutaneous fat area in men was the sole body composition trend associated with lung cancer incidence. The association also was not very strong with a 95% CI of 1.02–1.39 times higher risk. Therefore, the weakness of the association may have prevented a significant improvement in model fit for lung cancer incidence. In the context of lung cancer–specific mortality, loss of subcutaneous fat was significant in men, whereas in women, both loss of muscle area and subcutaneous fat were associated with increased lung cancer-specific mortality risk. The absence of improved model fit in some settings may therefore reflect sex-specific differences in the breadth and strength of associations between body composition trends and clinical outcomes.

### Potential future directions

Lung cancer screening currently focusses on nodule detection and lung cancer mortality prevention only and the screening pattern (i.e., interval and number of screenings) is not highly personalised. Knowing which biomarkers and biomarker trends are associated with increased risk of lung cancer mortality and which markers are possibly signs of underlying ‘competing’ disease could help personalise the interval at which subjects are screened for lung cancer. Body composition measurements could be included as one of those biomarkers. Other potential biomarkers are under investigation as part of the NELSON-POP project, such as genetics, air pollution, coronary artery calcium, and emphysema. An advantage of body composition over measurements of coronary artery calcium and emphysema is a lower susceptibility to movement and radiodensity measurement noise artefacts.

Aside from the long-term body composition trends we investigated, short-term body composition changes may hold additional information. Lung cancer incidence may cause changes in body composition, which in turn may impact mortality. Future research should aim to clarify the complex causal pathways between body composition and lung cancer. While baseline body composition may serve as a predictor for lung cancer incidence, short-term body composition changes during lung cancer follow-up might follow a different trajectory and provide additional information. Additionally, future studies could further analyse baseline body composition, body composition at each CT scan, and body composition changes separately in the context of lung cancer screening. This would include determining whether screening some subjects more often based on body composition as well as other biomarkers could improve outcomes. For example, as we found that low baseline subcutaneous fat area and low muscle density was associated with increased risk of lung cancer incidence, subjects matching those biomarkers could be screened more often. Future research may find that subjects that do not match these biomarkers could instead be screened less often without an increase in risk, possibly increasing the cost-effectiveness of lung cancer screening.

Additionally, if further research can demonstrate that the associations we found between body composition and all-cause mortality are causal, management of body composition and its trends could potentially improve patient outcomes. If a patient is losing muscle, exercise or a healthier diet can be employed to reduce this loss and in turn improve the outcome similar to prehabilitation.^7^ As only seventeen percent of the people in this study died of lung cancer, there is potential for a greater mortality reduction. This may be achievable by broadening the adverse outcomes to be prevented beyond lung cancer.

Both screening optimisation and mortality reduction require further studies to investigate whether there is additional value in incorporating body composition trends into risk models for personalised screening and whether it is beneficial to improve body composition in participants screened for lung cancer.

### Strengths and limitations

Our study has several strengths. First, it was based on a large screening cohort with substantial follow-up duration, providing sufficient power for stratified analyses. Second, the NELSON study design with four screening rounds over 5 years enabled reliable measurements of longitudinal trends in body composition.

However, our study also had some limitations. First, subjects who were diagnosed with lung cancer left the NELSON trial to undergo treatment, reducing the number of scans available for these subjects.^24^ Second, our study population included fewer women than men, weakening the confidence in the results for women. Third, our results are only applicable to a lung cancer screening population with long smoking history, limiting the generalisability to persons who have not smoked or smoked less and to those with symptoms who present outside a screening setting. Fourth, we only obtained a 2D single slice measurement of body composition, instead of a 3D body composition measurement of the whole thorax. Fifth, we used muscle density as a proxy for intramuscular fat, instead measuring intramuscular fat directly. Sixth, given the absence of an intervention, definite causal conclusions cannot be made despite adjustment for several variables other than body composition. Seventh, the inclusion criteria that required subjects to have at least two scans with at least 800 days between the first and last scan means that the found hazard ratios only apply to screening-adherent lower-risk individuals, as non-adherent or higher-risk individuals are less likely to meet those requirements. Eighth, although ordinary least squares linear models proved to provide evidence on the effect of body composition change on outcomes, they may not be suited to individual risk estimation due to the remaining unexplained variance introducing uncertainty. Finally, the models did not adjust for all possible influences on mortality, such as pulmonary or cardiac comorbidities. Therefore some degree of residual confounding cannot be excluded.

### Conclusion

We used a machine learning method to determine body composition metrics on repeated low-dose chest CT scans in a lung cancer screening population with long-term follow-up. Lower baseline skeletal muscle area and radiodensity, lower subcutaneous fat area, and decreases in area and radiodensity over time increased the risk of all-cause mortality, lung cancer incidence, and lung cancer-specific mortality. Including not just baseline body composition but also body composition change over time resulted in better risk models. Opportunistic evaluation of body composition on low-dose chest CT may add value to prediction of outcomes in lung cancer screening programs and may provide a target for intervention.

## Contributors

Ir. Stijn Bunk: Conceptualisation, Methodology, Formal analysis, Writing–Original Draft.

Dr. Ir. Edwin Bennink: Conceptualisation, Methodology, Resources, Data Curation, Writing–Review & Editing, Supervision.

Dr. Grigory Sidorenkov: Validation, Writing–Review & Editing.

Marjolein A. Heuvelmans: Writing–Review & Editing.

Prof. Dr. Harry Groen: Writing–Review & Editing.

Dr. Hester Gietema: Writing–Review & Editing.

Prof. Dr. Mathias Prokop: Writing–Review & Editing.

Prof. Dr. Joachim G. Aerts: Writing–Review & Editing.

Dr. Ir. Colin Jacobs: Writing–Review & Editing.

Prof. Dr. Geertruida de Bock: Writing–Review & Editing.

Prof. Dr. Pim A. de Jong: Conceptualisation, Methodology, Writing–Review & Editing, Supervision.

Prof. Dr. Rozemarijn Vliegenthart: Conceptualisation, Methodology, Writing–Review & Editing, Supervision, Funding acquisition.

Dr. Firdaus Mohamed Hoesein: Conceptualisation, Methodology, Writing–Review & Editing, Supervision.

All authors have read and approved the final version of the manuscript.

Stijn Bunk and Grigory Sidorenkov have accessed and verified the data reported in this manuscript.

## Data sharing statement

The datasets generated and/or analysed during the current study are not publicly available due to privacy concerns, but are accessible via the NELSON Data Access Board (https://umcgresearchdatacatalogue.nl/all/cohorts/NELSON) on reasonable request, after approval of a proposal and with a signed data access agreement.

## Declaration of generative AI and AI-assisted technologies in the manuscript preparation process

During the preparation of this work the author(s) used ChatGPT GPT-5 in order to provide suggestions for improving the readability of some paragraphs. After using this tool/service, the author(s) reviewed and edited the content as needed and take(s) full responsibility for the content of the published article.

## Declaration of interests

**MP** declares receiving institutional funding grants not related to the current article from the Dutch Cancer Society (AMARA, KWF 2021-14113) and from the European Commission (SOLACE, EU4 Health Programme 101101187), and membership as Vice President of the European Society of Radiology. **JGA** declares receiving funding grants from AstraZeneca, Danone, Genmab, and Amphere, consulting fees from CureVac, MSD, Amphera, AstraZeneca, and Accord, receiving presentation fees from BMS, MSD, and Eli-Lilly, being issued patents on allogenic tumour cell lysate, a combination immuneoncology drug and biomarker for immunotherapy, participation on a Data Safety Monitoring Board or Advisory board for AstraZeneca, MSD, Danone, CureVac, Genmab, Accord, and Vivace, membership as treasurer of the IASLC, being a board member of iMig, and having stock or stock option in trust in Amphera. **CJ** declares receiving research funding grants from MeVis Medical Solutions, Bremen, Germany, Siemens Healthineers, Erlangen, Germany, Philips Medical Systems, Best, Netherlands, royalties to the host institution for the product Veolity from Mevis Medical Solutions, Bremen, Germany, speaker fees from Canon Medical Systems for presenting at the ESCR-ESTI conference and from Johnson & Johnson for a symposium. **PAJ** declares the department of Radiology at the UMC Utrecht receives research support from Philips Healthcare. **RV** declares receiving grants not related to the current work from the Dutch Heart Foundation and institutional research grants from Siemens Healthineers, speaking fees from Bayer Healthcare, Siemens Healthineers, and Keya medical, membership of the scientific advisory board of the Lifelines organisation of the international advisory board of the Institute for cardiometabolism and nutrition, being the direct past president of the European Society of Cardiovascular Radiology, and membership of the editorial board of Radiology.

The other authors of this manuscript declare no relationships with any companies, whose products or services may be related to the subject matter of the article.

## Appendix A Supplementary data

Appendix A–D

Formal_acceptance_and_moved_to_production_ref_28
