# Supplementary material for: CT-based body composition and its change through time in relation to outcomes in participants screened for lung cancer
Source: eBioMedicine. 2026 Apr 30;127:106276. doi: 10.1016/j.ebiom.2026.106276 (PMC13145877; doi:10.1016/j.ebiom.2026.106276)
Supplement: Appendix A–D [file mmc1.docx]

**Appendices**

# Appendix A: Evaluating non-linear relationships

We evaluated the presence of non-linear relationships between body composition and outcomes using three additional Cox regression models. These Cox regression models used restricted cubic splines with four degrees of freedom for the body composition variables to model any non-linear relationship between the body composition variables and outcomes. We used the R packages *survival* and *SmoothHR* to analyse our data. The adjusted hazard ratios for all-cause mortality, lung cancer incidence, and lung cancer mortality relative to the body composition measurements for muscle and SAT are presented in Figure A1, Figure A2, and Figure A3, respectively. The reference level shown is the 50th percentile. If the grey confidence interval visualization does not cross over the 1.0-line, then the hazard ratio is significantly different from the reference level (P<0.05). The hazard ratios and 95% confidence intervals at the body composition metric mean plus and minus one standard deviation are reported in Table A1. These values are used in the following paragraphs as reference. The added value to model performance of splines is quantified in Table A2, showing a very modest performance increase.

### All-cause mortality

Firstly, we found that SAT baseline area was not associated with all-cause mortality risk at –SD and +SD, with the aHR at 89.1 cm^2^ being 1.22 (95%CI 0.95-1.33) and at 236.5 cm^2^ being 0.95 (0.78-1.16). However, at SAT baseline area values below 60 cm^2^ there was an increased all-cause mortality risk. Additionally, decreasing SAT area over time was associated with all-cause mortality risk, with an aHR at -5.4 cm²/year of 1.24 (1.11-1.38). Increasing SAT area over time was not significantly associated with an increase in all-cause mortality risk, with an aHR at 10.2 cm²/year of 0.94 (0.84-1.06).

Secondly, we found that a lower muscle baseline area was associated with an increase in all-cause mortality risk, with an aHR at 179.1 cm^2^ of 1.40 (1.14-1.74), whereas a higher muscle baseline was not significantly associated with all-cause mortality risk, with an aHR at 225.2 cm^2^ of 0.85 (0.71-1.02). Additionally, decreasing muscle area over time was associated with an increase in all-cause mortality risk, with an aHR at -5.6 cm²/year of 1.25 (1.13-1.38), and increasing muscle area over time was associated with a decrease in all-cause mortality risk, with an aHR at 2.3 cm²/year of 0.89 (0.79-1.00), though at higher values of muscle area increase over time the relationship loses significance.

Finally, we found that a lower baseline muscle radiodensity, indicating fatty muscle, was associated with an increase in all-cause mortality risk, with an aHR at 22.8 HU of 1.33 (1.11-1.60). A higher baseline muscle radiodensity was associated with a decrease in all-cause mortality risk, with an aHR at 34.0 HU of 0.81 (0.68-0.98). Additionally, a decrease in muscle radiodensity over time, indicating a decrease of muscle quality, was associated with an increase in all-cause mortality risk, with an aHR at -1.3 HU/year of 1.23 (1.09-1.40). Increasing radiodensity over time was not associated with all-cause mortality risk, with an aHR at 0.3 HU/year of 0.95 (0.82-1.09).

### Lung cancer incidence

Firstly, we found that a higher SAT baseline area was associated with an increase in lung cancer incidence risk, with an aHR at 89.1 cm^2^ of 1.73 (1.30-2.30), and a lower SAT baseline area was associated with a decrease in lung cancer incidence risk, with an aHR at 236.5 cm^2^ of 0.68 (0.47-0.98). Additionally, decreasing SAT area over time was associated with an increase in lung cancer incidence risk, with an aHR at -5.4 cm²/365 days of 1.36 (1.11-1.65). Increasing SAT area over time was not associated with lung cancer incidence risk, with an aHR at 10.2 cm²/365 daysof 0.98 (0.79-1.21).

Secondly, we found that muscle baseline area does not affect lung cancer incidence risk, with an aHR at 179.1 cm^2^ of 1.14 (0.75-1.73), and an aHR at 225.2 cm^2^ of 1.18 (0.85-1.64). Additionally, change in muscle area does not affect lung cancer incidence risk, with an aHR at -5.6 cm²/365 days of 1.11 (0.89-1.40), and an aHR at 2.3 cm²/365 days of 0.95 (0.73-1.23).

Finally, we found that a lower baseline muscle radiodensity, indicating fatty muscles, was associated with an increase in lung cancer incidence risk, with an aHR at 22.8 HU of 1.57 (1.13-2.19). A higher baseline muscle radiodensity was not associated with lung cancer incidence risk, with an aHR at 34.0 HU of 0.78 (0.56-1.08). Additionally, change in muscle radiodensity, indicating a change of muscle quality, was not associated with lung cancer incidence risk, with an aHR at -1.3 HU/365 days of 1.19 (0.92-1.54), and with an aHR at 0.3 HU/365 days of 0.77 (0.58-1.01).

### Lung cancer mortality

Firstly, we found that a lower SAT baseline area was associated with an increase in lung cancer mortality risk, with an aHR at 89.1 cm^2^ of 1.57 (1.07-2.30). A higher SAT baseline area was not associated with lung cancer mortality risk, with an aHR at 236.5 cm^2^ of 0.87 (0.54-1.39). Additionally, a decrease in SAT area over time was associated with an increase in lung cancer mortality risk, with an aHR at -5.4 cm²/365 days of 1.57 (1.19-2.08). An increase in SAT area over time was not associated with mortality risk, with an aHR at 10.2 cm²/365 days of 0.92 (0.67-1.26).

Secondly, we found that a lower muscle baseline area was not associated with lung cancer mortality risk, with an aHR at 179.1 cm^2^ of 1.06 (0.59-1.91), but a higher muscle baseline area increased lung cancer mortality risk, with an aHR at 225.2 cm^2^ of 1.59 (1.04-2.43). Additionally, a decrease in muscle area over time was not associated with lung cancer mortality risk, with an aHR at -5.6 cm²/365 days of 1.11 (0.81-1.52), but an increase in muscle area over time was associated with a decrease in lung cancer mortality risk, with an aHR at 2.3 cm²/365 days of 0.66 (0.45-0.99).

Finally, we found that a lower baseline muscle radiodensity was associated with an increase in lung cancer mortality risk, with an aHR at 22.8 HU of 2.08 (1.34-3.22). A higher baseline muscle radiodensity was not associated with lung cancer mortality risk, with an a HR at 34.0 HU of 0.75 (0.48-1.18). Lung cancer radiodensity change over time was not associated with with lung cancer mortality risk, with an aHR at -1.3 HU/365 days of 1.03 (0.72-1.47), and with and aHR at 0.3 HU/365 days of 0.78 (0.54-1.12).

| **Table A1. Adjusted Hazard ratios at selected body composition metric values** | | | | | | |
| --- | --- | --- | --- | --- | --- | --- |
|  | All-cause Mortality | | Lung Cancer Incidence | | Lung Cancer Mortality | |
|  | aHR | P-value | aHR | P-value | aHR | P-value |
| Age (Years) | **1.08 (1.06, 1.09)** | **<0.001** | **1.05 (1.02, 1.07)** | **<0.001** | **1.05 (1.02, 1.07)** | **0.002** |
| Female | **0.42 (0.27, 0.64)** | **<0.001** | 1.55 (0.80, 3.00) | 0.200 | 1.96 (0.79, 4.83) | 0.140 |
| Currently smoking | **1.55 (1.33, 1.82)** | **<0.001** | **1.49 (1.12, 1.99)** | **0.007** | **2.34 (1.56, 3.51)** | **<0.001** |
| Pack-years | **1.01 (1.00, 1.01)** | **<0.001** | **1.01 (1.00, 1.01)** | **<0.001** | **1.02 (1.01, 1.02)** | **<0.001** |
|  | Mean-SD aHR | Mean+SD aHR | Mean-SD aHR | Mean+SD aHR | Mean-SD aHR | Mean+SD aHR |
| Baseline subcutaneous fat [89.1, 236.5] (cm²) | 1.12 (0.95, 1.33) | 0.95 (0.78, 1.16) | **1.73 (1.30, 2.30)** | **0.68 (0.47, 0.98)** | **1.57 (1.07, 2.30)** | 0.87 (0.54, 1.39) |
| Subcutaneous fat trend [-5.4, 10.2] (cm²/365 days) | **1.24 (1.11, 1.38)** | 0.94 (0.84, 1.06) | **1.36 (1.11, 1.65)** | 0.98 (0.79, 1.21) | **1.57 (1.19, 2.08)** | 0.92 (0.67, 1.26) |
| Baseline muscle area [179.1, 255.2] (cm²) | **1.40 (1.14, 1.74)** | 0.85 (0.71, 1.02) | 1.14 (0.75, 1.73) | 1.18 (0.85, 1.64) | 1.06 (0.59, 1.91) | **1.59 (1.04, 2.43)** |
| Muscle area trend [-5.6, 2.3] (cm²/365 days) | **1.25 (1.13, 1.38)** | **0.89 (0.79, 1.00)** | 1.11 (0.89, 1.40) | 0.95 (0.73, 1.23) | 1.11 (0.81, 1.52) | **0.66 (0.45, 0.99)** |
| Muscle density [22.8, 34.0] (HU) | **1.33 (1.11, 1.60)** | **0.81 (0.68, 0.98)** | **1.57 (1.13, 2.19)** | 0.78 (0.56, 1.08) | **2.08 (1.34, 3.22)** | 0.75 (0.48, 1.18) |
| Muscle density trend [-1.3, 0.3] (HU/365 days) | **1.23 (1.09, 1.40)** | 0.95 (0.82, 1.09) | 1.19 (0.92, 1.54) | 0.77 (0.58, 1.01) | 1.03 (0.72, 1.47) | 0.78 (0.54, 1.12) |
| Presented hazard ratios have been adjusted for confounders. Values between square brackets are the Mean-SD and the Mean+SD respectively. Values between parentheses are the 95%CI hazard ratios. Significant results bolded. aHR = Adjusted Hazard Ratio. SD = Standard Deviation. HU = Hounsfield Unit. | | | | | | |

| **Table A2. Added value of splines in Cox models for all-cause mortality, lung cancer incidence, and lung cancer mortality.** | | | | |
| --- | --- | --- | --- | --- |
|  | Model performance | | | |
|  | Without splines | | With splines | |
|  | C-Statistic | AIC (DF) | C-Statistic | AIC (DF) |
| All-cause mortality | 0.68 (0.67, 0.70) | 12652.61 (10) | 0.7 (0.68, 0.72) | 12607.65 (28.04212) |
| Lung cancer incidence | 0.66 (0.62, 0.69) | 3889.304 (10) | 0.68 (0.65, 0.72) | 3885.228 (27.88956) |
| Lung cancer mortality | 0.71 (0.67, 0.76) | 2141.484 (10) | 0.75 (0.71, 0.79) | 2140.485 (27.91365) |
| Values between parentheses are the 95% confidence interval. AIC = Akaike Information Criterion. DF = Degrees of Freedom. | | | | |





**Figure A1. Hazard ratio graphs for the relationship of all-cause mortality with a) SAT baseline area, b) SAT area change rate, c) muscle baseline area, d) muscle area change rate, e) muscle baseline radiodensity, and f) muscle radiodensity change rate. The reference level shown is the 50th percentile. If the grey 95% confidence interval visualization does not cross over the dashed 1.0-line, then the hazard ratio is significantly different from the reference level (p<0.05). The histogram indicates the number of subjects falling within that bin of the values shown on the x-axis. The upper and lower 2 percent of subjects are not plotted.**





**Figure A2. Hazard ratio graphs for the relationship of lung cancer incidence with a) SAT baseline area, b) SAT area change rate, c) muscle baseline area, d) muscle area change rate, e) muscle baseline radiodensity, and f) muscle radiodensity change rate. The reference level shown is the 50th percentile. If the grey 95% confidence interval visualization does not cross over dashed the 1.0-line, then the hazard ratio is significantly different from the reference level (p<0.05). The histogram indicates the number of subjects falling within that bin of the values shown on the x-axis. The upper and lower 2 percent of subjects are not plotted.**





**Figure A3. Hazard ratio graphs for the relationship of lung cancer incidence with a) SAT baseline area, b) SAT area change rate, c) muscle baseline area, d) muscle area change rate, e) muscle baseline radiodensity, and f) muscle radiodensity change rate. The reference level shown is the 50th percentile. If the grey 95% confidence interval visualization does not cross over the 1.0-line, then the hazard ratio is significantly different from the reference level (p<0.05). The histogram indicates the number of subjects falling within that bin of the values shown on the x-axis. The upper and lower 2 percent of subjects are not plotted.**

# Appendix B: Low-dose CT scan number distribution

| **Table B1. Number of scans per subject** | | | | | | |
| --- | --- | --- | --- | --- | --- | --- |
| Number of scans, n | 2 | 3 | 4 | 5 | 6 | 7-10 |
| Subjects overall, n (%) | 118 (1.9%) | 1519 (24.6%) | 3394 (54.9%) | 904 (14.6%) | 198 (3.2%) | 54 (0.9%) |
| Subjects who died, n (%) | 20 (2.6%) | 261 (34.5%) | 327 (43.2%) | 108 (14.3%) | 30 (4.0%) | 11 (1.4%) |
| Subjects with lung cancer, n (%) | 4  (1.6%) | 60 (23.4%) | 111 (43.4%) | 47 (18.4%) | 24 (9.4%) | 10 (3.9%) |
| Subjects who died of lung cancer, n (%) | 2  (1.6%) | 28 (21.9%) | 62 (48.4%) | 21 (16.4%) | 9  (7.0%) | 6 (4.7%) |

# Appendix C: Error check of dataset

**Protocol for error check of body composition measurements.**

Three different methods were used to select subjects for which the output was evaluated for errors.

1. Random sample of 200 subjects.
2. 200 subjects of which at least one scan is in the 99.1% percentile of SAT or muscle.
3. 200 subjects of which the standard deviation of the ratio of the areas relative to T5 across all scan moments of the subject was in the 99.1% percentile.

The groups received the following labels.

1. Output has no error.
2. Output has small error (<10% area incorrect), still usable.

This includes:

1. Small holes in the segmentation due to errors by the segmentation method
2. Small amounts of image noise resulting in incorrect segmentation.
3. Small amounts of incorrectly identified tissue.
4. Output has large error (>%10 area incorrect), not usable.

This includes:

1. Large holes in the segmentation due to errors by the segmentation method.
2. Large amounts of image noise resulting in incorrect segmentation.
3. Large amounts of incorrectly identified tissue.
4. Corrupt images.

The number of images for each type of label are reported in Table C1. The number of large errors in the dataset was relatively small.

| **Table C1. Error rates detected in each of the three different samples.** | | | | | | |
| --- | --- | --- | --- | --- | --- | --- |
| Error Type | Muscle T5 | Muscle T8 | Muscle T10 | SAT T5 | SAT T8 | SAT T10 |
| Random sample | | | | | | |
| No error | 522 | 488 | 516 | 580 | 554 | 565 |
| Small error | 170 | 223 | 187 | 135 | 165 | 147 |
| Large error | 27 | 8 | 16 | 4 | 0 | 7 |
| No error (%) | 72.6 | 67.9 | 71.8 | 80.7 | 77.1 | 78.6 |
| Small error (%) | 23.6 | 31 | 26 | 18.8 | 22.9 | 20.4 |
| Large error (%) | 3.8 | 1.1 | 2.2 | 0.6 | 0 | 1 |
| Percentile sample | | | | | | |
| No error | 480 | 471 | 492 | 590 | 497 | 523 |
| Small error | 269 | 304 | 273 | 200 | 299 | 255 |
| Large error | 66 | 40 | 50 | 25 | 19 | 37 |
| No error (%) | 58.9 | 57.8 | 60.4 | 72.4 | 61 | 64.2 |
| Small error (%) | 33 | 37.3 | 33.5 | 24.5 | 36.7 | 31.3 |
| Large error (%) | 8.1 | 4.9 | 6.1 | 3.1 | 2.3 | 4.5 |
| Ratio sample | | | | | | |
| No error | 437 | 423 | 484 | 507 | 458 | 507 |
| Small error | 250 | 306 | 250 | 269 | 327 | 265 |
| Large error | 117 | 75 | 70 | 28 | 19 | 32 |
| No error (%) | 54.4 | 52.6 | 60.2 | 63.1 | 57 | 63.1 |
| Small error (%) | 31.1 | 38.1 | 31.1 | 33.5 | 40.7 | 33 |
| Large error (%) | 14.6 | 9.3 | 8.7 | 3.5 | 2.4 | 4 |
| T5/T8/T10 = measurement was obtained at the 5th/8th/10th thoracic vertebrae respectively. SAT = Subcutaneous adipose tissue. | | | | | | |

**Appendix D: Population characteristics of included and excluded participants**

| **Table D1. Population characteristics, overall and by sex for included and excluded populations.** | | | | | | |
| --- | --- | --- | --- | --- | --- | --- |
|  | Included | | | Excluded | | |
| Characteristic | Overall (n=6187) | Male (n=5228) | Female (n=959) | Overall (n=870) | Male (n=697) | Female (n=173) |
| Age (year) | 58.6 ± 5.5 | 58.8 ± 5.4 | 57.8 ± 5.5 | 60.0 ± 6.3 | 60.4 ± 6.3 | 58.4 ± 5.9 |
| Currently smoking | 3408 (55.1%) | 2864 (54.8%) | 544 (56.7%) | 490 (56.3%) | 386 (55.4%) | 104 (60.1%) |
| Pack-years | 41.2 ± 18.3 | 41.3 ± 18.6 | 40.5 ± 16.4 | 44.5 ± 34.6 | 45.7 ± 37.8 | 39.9 ± 15.2 |
| Follow-up years (IQR) | 12.2 (1.2) | 12.2 (1.0) | 11.4 (0.2) | 11.4 (6.5) | 11.4 (7.7) | 11.4 (0.4) |
| Deaths | 757 (12.2%) | 685 (13.1%) | 72 (7.5%) | 349 (40.1%) | 316 (45.3%) | 33 (19.1%) |
| Lung cancer cases | 256 (4.1%) | 219 (4.2%) | 37 (3.9%) | 169 (19.4%) | 136 (19.5%) | 33 (19.1%) |
| Lung cancer deaths | 128 (2.1%) | 109 (2.1%) | 19 (2.0%) | 76 (8.7%) | 67 (9.6%) | 9 (5.2%) |
| Baseline to final scan time (years) | 5.0 ± 1.1 | 5.0 ± 1.1 | 4.9 ± 1.1 | 0.9 ± 1.0 | 0.8 ± 0.9 | 0.9 ± 1.1 |
| The number of excluded participants reported in this table is 20 lower than in Figure 3 in the main manuscript due to missing characteristics. | | | | | | |
