# Supplementary material for: CT-based body composition and its change through time in relation to outcomes in participants screened for lung cancer
Source: eBioMedicine. 2026 Apr 30;127:106276. doi: 10.1016/j.ebiom.2026.106276 (PMC13145877; doi:10.1016/j.ebiom.2026.106276)
Supplement: Formal_acceptance_and_moved_to_production_ref_28 [file mmc2.pdf]

Outlook

Notification of Formal Acceptance for PONE-D-25-45870R1 - [EMID:4202dd96a739b36b]

Van em.pone.0.9aa251.c2bb4af8@editorialmanager.com <em.pone.0.9aa251.c2bb4af8@editorialmanager.com> namens PLOS ONE <em@editorialmanager.com>

Datum Do 9-4-2026 05:07

Aan Bunk-2, S.A.O. (Stijn) <S.A.O.Bunk-2@umcutrecht.nl>

CC: h.e.bennink-2@umcutrecht.nl, g.sidorenkov@umcg.nl, hester.gietema@mumc.nl, h.j.m.groen@umcg.nl, g.h.de.bock@umcg.nl, p.dejong-8@umcutrecht.nl, r.vliegenthart@umcg.nl, f.a.a.mohamedhoesein@umcutrecht.nl

PONE-D-25-45870R1
PLOS One

Dear Dr. Bunk,

I'm pleased to inform you that your manuscript has been deemed suitable for publication in PLOS One. Congratulations! Your manuscript is now being handed over to our production team.

At this stage, our production department will prepare your paper for publication. This includes ensuring the following:

* All references, tables, and figures are properly cited
* All relevant supporting information is included in the manuscript submission,
* There are no issues that prevent the paper from being properly typeset

You will receive further instructions from the production team, including instructions on how to review your proof when it is ready. Please keep in mind that we are working through a large volume of accepted articles, so please give us a few days to review your paper and let you know the next and final steps.

Lastly, if your institution or institutions have a press office, please let them know about your upcoming paper now to help maximize its impact. If they'll be preparing press materials, please inform our press team within the next 48 hours. Your manuscript will remain under strict press embargo until 2 pm Eastern Time on the date of publication. For more information, please contact onepress@plos.org.

You will receive an invoice from PLOS for your publication fee after your manuscript has reached the completed accept phase. If you receive an email requesting payment before acceptance or for any other service, this may be a phishing scheme. Learn how to identify phishing emails and protect your accounts at https://eur05.safelinks.protection.outlook.com/?url=https%3A%2F%2Fexplore.plos.org%2Fphishing&data=05%7C02%7Cs.a.o.bunk-2%40umcutrecht.nl%7C47df634d525246cc822f08de95e525d2%7Cdcdf4a3dd0c04a6394cf781981249be5%7C0%7C0%7C639113008576540050%7CUnknown%7CTWFpbGZsb3d8eyJFbXB0eU1hcGkiOnRydWUsIlYiOiIwLjAuMDAwMCIsIlAiOiJXaW4zMiIsIkFOIjoiTWFpbCIsIldUIjoyfQ%3D%3D%7C0%7C%7C%7C&sdata=

bu5cMEFh%2BQAFBtB2Tqa1ip7X0QCdW7bafOheKj9VdJU%3D&reserved=0.

If we can help with anything else, please email us at customercare@plos.org.

Thank you for submitting your work to PLOS ONE and supporting open access.

Kind regards,
PLOS ONE Editorial Office Staff

on behalf of
Prof. Zhentian Wang
Academic Editor
PLOS One

In compliance with data protection regulations, you may request that we remove your personal registration details at any time. (Use the following URL: https://eur05.safelinks.protection.outlook.com/?url=https%3A%2F%2Fwww.editorialmanager.com%2Fpone%2Flogin.asp%3Fa%3Dr&data=05%7C02%7Cs.a.o.bunk-2%40umcutrecht.nl%7C47df634d525246cc822f08de95e525d2%7Cdcdf4a3dd0c04a6394cf781981249be5%7C0%7C0%7C639113008576583663%7CUnknown%7CTWFpbGZsb3d8eyJFbXB0eU1hcGkiOnRydWUsIlYiOiIwLjAuMDAwMCIsIlAiOiJXaW4zMiIsIkFOIjoiTWFpbCIsIldUIjoyfQ%3D%3D%7C0%7C%7C%7C&sdata=FeYJOY2kWG1lBYLdo2P3OVjGMBMrmQyojBppgdKPK38%3D&reserved=0). Please contact the publication office if you have any questions.

Outlook

Your manuscript has moved into production: [PONE] / [10.1371/journal.pone.0332004] / PONE-D-25-45870

Van pone@newgen.co <pone@newgen.co>
Datum Do 9-4-2026 16:32
Aan Bunk-2, S.A.O. (Stijn) <S.A.O.Bunk-2@umcutrecht.nl>

U ontvangt niet vaak e-mail van pone@newgen.co. Ontdek waarom dit belangrijk is

Dear Ir.Stijn Bunk,

Manuscript ID: PONE-D-25-45870

We are pleased to inform you that your manuscript, entitled "Repeatability of automated body composition measurement on low dose chest CT in male subjects," submitted to PONE, has moved into production. This transition marks the next step following the recent acceptance of your paper. Please note that this email is for your information only, and no response is required.

What to Expect Next:

As your paper progresses through the production process, it will undergo the following key milestones:

1. **Manuscript Completeness Check**
   Our team will ensure all elements of your manuscript are complete and adhere to the journal's submission guidelines.
2. **Style Editing**
   Professional editors will refine your manuscript for consistency, clarity, and adherence to our style standards.
3. **Proof Review**
   You will receive a proof of your manuscript for final review. This is your opportunity to verify the accuracy of the content and suggest any necessary corrections.
4. **Paper Ready for Publishing**
   Once final corrections are made, your paper will be prepared for online publication.
5. **Paper Published**
   Your paper will be published online and available to the academic community and the public.

Please keep an eye on your email for the proof review stage, as your timely review and approval are crucial to ensure accurate and prompt publication. We appreciate your contribution and look forward to seeing your work published.

Thank you for choosing PONE.

Best regards,

Vivek

PONE

This email was sent from Pubkit mailing system by pone@newgen.co.
